# Evaluation of Cardiac Substructures Dose Sparing in Single and Dual Isocenter RapidArc™ Radiotherapy Planning for Synchronous Bilateral Breast Cancer

**DOI:** 10.7759/cureus.48247

**Published:** 2023-11-04

**Authors:** Jahnabi Das, Shantanu K Mishra, Moirangthem N Singh, Mouchumee Bhattacharyya, Yanpothung Yanthan, Apurba K Kalita

**Affiliations:** 1 Radiation Oncology, Dr. Bhubaneswar Borooah Cancer Institute, Guwahati, IND

**Keywords:** dual isocentric rapidarc, mono isocentric rapidarc, avoidance sector, left anterior descending artery, synchronous bilateral breast cancer

## Abstract

Purpose

This study compares the dosimetry and dose sparing of cardiac substructures in single isocenter and dual isocenter RapidArc™ (Varian Medical Systems, Palo Alto, California, United States) radiotherapy planning for synchronous bilateral breast cancer.

Methodology

Six synchronous bilateral breast cancer (SBBC) patients received adjuvant radiation with the prescribed dose of 40.05 Gy in 15 fractions to the planning target volume (PTV) without local lymph nodal regions. PTVs and organs at risk (OARs), including both lungs, esophagus, spinal cord, heart, and left anterior descending coronary artery (LAD), both atria and ventricles were contoured. Single isocentric RapidArc (SIRA) and dual isocentric RapidArc (DIRA) plans were made for each patient and dosimetric differences between these two techniques were evaluated.

Results

There was no statistically significant difference in conformity index (CI) values between SIRA and DIRA plans, with 0.9681±0.01 and 0.9721±0.01 (p=0.505), respectively. SIRA planning showed superior homogeneity with homogeneity Index (HI) values of 0.0999±0.01 compared to DIRA planning with HI values of 0.1640±0.12 (p=0.230). The mean LAD dose of SIRA was valued higher than that of DIRA planning. Lower mean doses were obtained for both lungs in SIRA plans compared to DIRA plans. Meanwhile, doses to the right atrium, left atrium, left ventricle, right ventricle, and esophagus showed no statistical significance between these two techniques, except in the spinal cord.

Conclusion

Both SIRA and DIRA plans have satisfactory outcomes in sparing OARs. Meanwhile, SIRA techniques have less setup time and overall machine time.

## Introduction

Breast cancer is now one of the most commonly diagnosed cancers and the fifth leading cause of cancer mortality. According to Global Cancer Observatory (GLOBOCAN) 2020, female breast cancer accounts for 2.3 million (6.9%) cancer deaths [1]. Bilateral breast cancer (BBC) is becoming more common due to rising breast cancer incidence rates, better treatment options, and longer life expectancies. About 2-11% of all breast cancers are bilateral. For patients with unilateral breast cancer (UBC), the cumulative incidence rate of developing contralateral breast cancer at 10 years is approximately 3.4%. For women with a BRCA mutation, it is 13-40% [2]. BBC can be classified as synchronous BCC (SBBC) or metachronous BCC (MBBC), depending on how long it took for the first and second tumour diagnoses to occur. While MBBC demonstrated non-superior survival compared to UBC, BBC and SBBC demonstrated a worse prognosis than UBC. SBBC is described as two malignant tumours in each breast within six months [3].

There are no specific recommendations for managing BBCs [4]. There are numerous issues with radiation therapy in BBCs, including increased dose at the chest wall's core, increased total lung dose, and increased heart dose [5]. 

The three-dimensional (3D) conformal radiotherapy technique (CRT) approach with two tangential fields is frequently utilised in the treatment planning of unilateral breast cancer. While in SBBC, to minimise the overlapping region and maintain target coverage, a traditional tangential field with dual isocentres is laborious [4]. Inhomogeneity of the target coverage and poor cosmetic effects can be observed in 3DCRT. Compared to 3DCRT, intensity-modulated radiation therapy (IMRT)/volumetric modulated arc therapy (VMAT)/RapidArc™ (Varian Medical Systems, Inc., Palo Alto, California, United States) (RA) can improve the target dose coverage and achieve acceptable cosmetic effects of cardiopulmonary sparing. Using modern radiation therapy techniques, such as IMRT/ VMAT/RA, to treat both breasts simultaneously in SBBC patients is feasible, tolerable, and safe [6]. Using synchronous bilateral hypofractionated radiation (SBHRT) in SBBC patients with dual versus monocentric VMAT technique is rare, with few papers demonstrating its usefulness.

Cardiovascular disease following radiation therapy has surpassed breast cancer as the major cause of non-breast cancer death [5]. As a result, radiotherapy-induced cardiotoxicity is a significant issue that requires extensive research. Darbey et al. discovered a linear association between mean heart dose (MHD) and the incidence of ischemic heart disease, which rose by 7.4% for each Gy of MHD [7]. As a result, lowering the MHD is crucial for lowering long-term cardiotoxicity. Most studies have discovered that radiotherapy-induced cardiotoxicity is also closely related to the dose of key heart substructures, such as the left anterior descending artery (LAD) and the left ventricle because studies have shown that high-grade coronary stenosis in the LAD is increased in women receiving radiation for the breasts.

This observational retrospective study aimed to evaluate the clinicopathologic and dosimetric analysis of a single isocentric versus dual isocentric RA technique in SBBC patients treated with hypofractionated radiotherapy with heart and substructure sparing in a tertiary cancer centre in North East India.

## Materials and methods

This is a retrospective study that was conducted at Dr. Bhubaneswar Borooah Cancer Institute (BBCI), Guwahati, Assam, India. The Medical Ethics Committee, BBCI (Registration no: ECR/1040/Inst/AS/2018/RR-22) approved this study (approval number: 31/22).

Patient selection

Six patients diagnosed with SBBC who underwent treatment in BBCI with external radiotherapy between September 2020 and January 2023 were retrospectively studied. Table 1 shows the demographic and tumour characteristics of the six patients included in the study. The median age of the patients was 57 years.

**Table 1:** Patient characteristics BCS: breast conservation surgery; MRM: modified radical mastectomy; NACT: neoadjuvant chemotherapy; Adjct: adjuvant chemotherapy; ER: estrogen receptor; PR: progesterone receptor

		Patient 1	Patient 2	Patient 3	Patient 4	Patient 5	Patient 6
Age (In years)		67	57	50	48	57	60
Stage	Right	cT1N0M0	cT3N0M0	cT3N0M0	cT3N0M0	cT2N0M0	cT1N0M0
	Left	cT1N0M0	cT3N0M0	cT3N0M0	cT2N0M0	cT2N0M0	cT3N0M0
Surgery	BCS=1, MRM=2	1	2	2	2	1	Right=1; Left=2
Chemotherapy	NACT=1, Adjct=2	0	1	2	1	2	2
Hormone Status	ER	Positive	Positive	Positive	Positive	Positive	Positive
	PR	Positive	Positive	Negative	Positive	Negative	Positive
	HER 2NEU	Negative	Positive	Positive	Negative	Positive	Negative
Postoperative histology		Solid Pappilary Ca pTisN0	Right and left invasive ductal Ca ypT2N0; ypT0N0	Right IDC Grade 2 pT3N0 Left IDC Grade3 pT3N0	Right IDC Grade 2 Right ypT1N1; No tumour in left breast	Right IDC GRADE 3pT2N0M; Left IDC GRADE 3 pT2N0	Invasive Lobular Grade 2 Left: pT2N1 Right pT1aN0
ENE		Negative	Negative	Negative	Negative	Negative	Negative
LVSI		Negative	Negative	Negative	Negative	Negative	Negative

Simulation and contouring

All patients were immobilised in the breast board in a supine position with a 15˚ inclination angle. A planning computed tomography (CT) scan was done in a Philips Brilliance Big Bore CT simulator (Koninklijke Philips N.V., Amsterdam, Netherlands) without intravenous contrast. The acquisition was done with a slice thickness of 3 mm. The scan was imported into the Eclipse™ treatment planning system version 15.6 (Varian Medical Systems).

The clinical target volume (CTV) included the bilateral chest wall/bilateral breasts and post-modified radical mastectomy/breast conservative surgery. Planning target volume (PTV) breast/chest wall was generated by 5 mm isotropic expansion of the CTV. The PTV thus obtained was cropped by 3 mm to exclude the regions extending outside the body. The organs at risk (OARs) included the heart and its chambers, left anterior descending coronary artery (LAD), bilateral lungs, esophagus, and spinal cord, delineated following the Radiation Therapy Oncology Group (RTOG) recommendations. The average PTV was 1169±615 cc, ranging from 564.3 cc to 1939.3 cc.

RA planning

In this study, the same physicist created all the plans using the Varian Eclipse treatment planning system with 6 MV photon beams, with the prescribed radiation dose of 40.05 Gy in 15 fractions (for all cases, 95% of PTV should receive 95% of the prescribed dose). For each patient, two RA plans were created, one using the single isocentre (Single Isocentric RA (SIRA) technique) and the other using two isocentres (Dual Isocentric RA (DIRA) approach). In the SIRA technique, the isocentre was placed in the center of the body midway between the two breasts/chest walls. In the DIRA technique, one isocentre was placed close to the left-sided PTV, and the other isocentre was placed near the right-sided PTV. In the SIRA technique, four arcs were utilised for treatment planning. Out of the four arcs, in two arcs, the avoidance sector was placed across the lung region on both sides. In the DIRA technique, two arcs were utilised without any sectoral avoidance. The arc start and stop angles were selected as per the posterior extension of the target and it was not identical in all six patients. The typical value of the arc start angle was 150-210° in clockwise rotation and 210-150° in anti-clockwise rotation. However, for the same patient, the arc start angle for the SIRA plan was identical to the arc start angle for the DIRA plan. The plans were optimized using the photon optimizer (PO) algorithm version 15.6 (Varian Medical Systems). In both plans, the optimization objectives were kept the same. An anisotropic analytical algorithm (AAA) was used for the final dose calculation with a grid size of 2.5mm in all plans.

Plan evaluation and statistical analysis

All the plans were evaluated for the homogeneity index (HI) and conformity index (CI) of the PTV and the dosimetric parameters of OARs. CI [8] and HI [9] were calculated using the formulae below.

CI= V95%/PTV volume

HI= (D2%- D98%)/D50%

where V95%= volume of the isodose of 95% of the prescribed dose; D2%, D50%, and D98% are doses to 2%, 50%, and 98% of the PTV, respectively.

OAR dose constraints of plans were based on department protocol obtained from the Quantitative Analyses of Normal Tissue Effects in the Clinic (QUANTEC) study [10].

Quantitative analysis of plans was done by dose volume histogram (DVH) analysis. IBM SPSS Statistics for Windows, Version 25.0 (Released 2017; IBM Corp., Armonk, New York, United States) was used to conduct the statistical research, with statistical significance defined as a p-value of 0.05.

## Results

**Figure 1:**
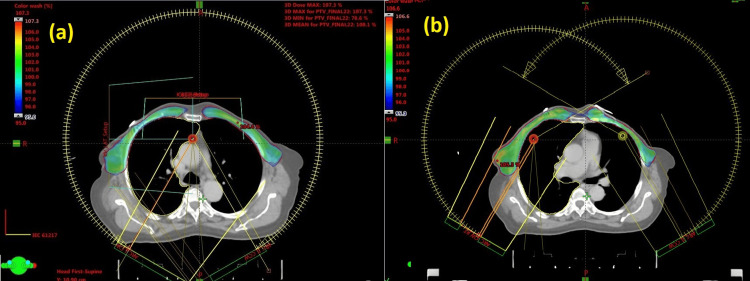
The 95% isodose distribution of (a) SIRA and (b) DIRA plans of bilateral breast cancer. SIRA: single isocentric RapidArc; DIRA: dual isocentric RapidArc

**Figure 2:**
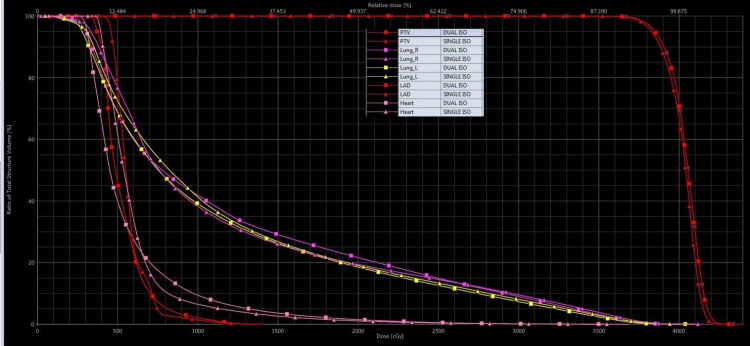
The dose volume histogram showing both the target volumes and organ at risks of SIRA plan (triangle) and DIRA plan (square) techniques DIRA: dual Isocentric RapidArc; SIRA: single isocentric RapidArc

Comparison of PTV and OAR doses using dosimetric parameters, including CI and HI of SIRA and DIRA plans of all patients, are presented in Table 2. Figure 1a illustrates a SIRA plan, and Figure 1b depicts a DIRA plan. The green dose colour wash represents 95% of the prescribed dose to the PTV. Figure 2 depicts the DVH of target volumes and OARs of both SIRA and DIRA.

**Table 2:** Comparison of PTV and OAR doses of SIRA and DIRA plans using dosimetric parameters PTV: planning target volume; Max: maximum dose; Mean: mean dose; V5%: volume of structure (in percentage) receiving at least 5Gy dose; V10%: volume of structure (in percentage) receiving at least 10 Gy; V20%: volume of structure (in percentage) receiving at least 20 Gy; V30%: volume of structure (in percentage) receiving  at least 30Gy; V107: Volume of structure in cc receiving at least 107% of the prescribed dose; CI: conformity index; HI: homogeneity index; OAR: organs at risk; DIRA: dual Isocentric RapidArc; SIRA: single isocentric RapidArc

Structure	Parameters	Single Isocentre Rapid Arc (SIRA)							Dual Isocentre Rapid Arc (DIRA)							P-value
		Pt. 1	Pt. 2	Pt. 3	Pt. 4	Pt. 5	Pt. 6	Average±SD	Pt. 1	Pt. 2	Pt. 3	Pt. 4	Pt. 5	Pt. 6	Average±SD	
PTV	CI	0.9670	0.9612	0.9645	0.9651	0.9776	0.9730	0.9681±0.01	0.9670	0.9668	0.9715	0.9987	0.9626	0.9659	0.9721±0.01	0.505
	HI	0.1036	0.0984	0.0973	0.0997	0.0870	0.1136	0.0999±0.01	0.0996	0.1010	0.0921	0.4107	0.1765	0.1040	0.1640±0.12	0.230
	V107 (cc)	0.14	0.00	0.00	0.00	0.00	0.20	0.06±0.09	0.01	0.00	0.06	0.11	0.02	0.05	0.04±0.04	0.710
Right Lung	V20(%)	3.50	18.30	21.40	19.40	14.80	3.60	13.50±8.00	13.50	27.20	29.00	28.40	25.00	3.80	21.15±10.25	0.180
	V30(%)	0.30	8.30	9.80	9.50	7.40	0.80	6.02±4.32	1.30	14.30	12.30	15.30	9.40	0.60	8.87±6.46	0.390
	Dmean (Gy)	9.64	12.95	13.94	11.94	10.83	8.00	11.22±2.19	9.77	12.53	14.32	14.64	13.75	7.20	12.04±2.96	0.590
Left Lung	V20(%)	1.80	11.10	24.50	19.00	13.80	15.00	14.20±7.66	11.30	22.00	27.10	27.50	14.60	13.20	19.28±7.19	0.260
	V30(%)	0.30	4.60	9.40	8.10	6.90	6.00	5.88±3.20	0.90	12.30	10.90	15.50	7.00	5.10	8.62±5.30	0.305
	Dmean (Gy)	9.64	10.03	14.88	11.84	10.02	11.00	11.24±1.96	9.99	12.46	14.13	14.93	10.62	10.57	12.12±2.06	0.450
Heart	V5(%)	43.70	57.30	75.30	61.50	38.20	67.30	57.22±14.08	50.00	71.80	57.90	76.40	80.40	50.60	64.52±13.38	0.420
	V10(%)	1.10	6.80	9.80	6.50	7.70	10.60	7.08±3.36	10.50	35.00	10.70	32.90	25.60	10.90	20.93±11.64	0.060
	Dmean (Gy)	5.27	6.02	6.84	6.12	5.73	6.20	6.03±0.52	5.84	7.50	6.28	9.24	8.51	6.29	7.28±1.38	0.130
LAD	V10%	3.60	34.10	35.50	10.40	7.90	8.20	16.62±14.26	41.90	43.40	19.20	23.80	31.10	0.00	26.57±16.17	0.170
	Dmean (Gy)	7.67	10.39	9.04	5.74	5.62	6.30	7.46±1.94	10.50	25.44	7.71	8.77	7.96	4.62	10.83±7.41	0.830
	Dmax Gy	15.72	35.75	25.70	14.08	21.86	9.21	20.39±9.52	25.95	39.29	26.34	21.76	20.62	7.48	23.57±10.31	0.580
Right Atrium	V5(%)	7.00	29.40	74.20	59.00	0.15	89.90	43.28±36.73	25.80	99.40	68.50	90.20	88.50	31.60	67.33±31.63	0.420
	Dmean (Gy)	4.02	4.87	5.87	5.34	4.34	5.78	5.04±0.76	4.42	11.58	6.76	7.07	6.70	4.89	6.90±2.54	0.060
	Dmax Gy	6.62	11.27	13.11	9.57	9.06	9.50	9.86±2.19	12.22	30.06	17.57	14.52	13.15	9.87	16.23±7.24	0.130
Left Atrium	V5(%)	33.40	16.70	27.00	47.30	0.00	3.00	21.23±18.25	6.40	58.10	32.20	34.40	39.00	0.00	28.35±21.61	0.170
	Dmean (Gy)	4.71	4.30	4.93	4.97	3.89	4.65	4.58±0.41	3.34	5.84	4.76	4.75	4.64	2.90	4.37±1.07	0.300
	Dmax Gy	9.43	7.29	8.55	7.05	5.22	6.23	7.30±1.53	5.83	13.64	7.14	7.95	8.00	3.82	7.73±3.30	0.580
Left Ventricle	V5(%)	16.10	65.80	87.10	41.20	34.70	99.80	57.45±32.35	30.20	97.50	50.30	91.90	93.40	56.30	69.93±28.08	0.490
	Dmean (Gy)	4.18	6.54	7.90	5.59	5.56	7.80	6.26±1.44	5.02	18.63	5.81	11.22	9.87	5.87	9.40±5.16	0.180
	Dmax Gy	14.30	36.99	30.15	34.54	30.12	33.05	29.86±8.06	25.57	40.39	30.07	37.01	34.92	31.82	33.30±5.27	0.402
Right Ventricle	V5(%)	21.10	80.50	70.80	56.20	76.30	100.00	67.48±26.81	39.10	99.70	53.70	100.00	96.90	86.40	79.30±26.37	0.450
	Dmean (Gy)	4.29	4.62	6.27	6.21	6.30	8.69	6.06±1.57	4.77	22.85	5.74	14.79	9.45	8.37	11.00±6.79	0.110
	Dmax Gy	7.27	34.85	16.65	24.31	29.12	31.98	24.03±10.41	12.35	40.74	17.92	38.57	32.22	30.60	28.73±11.33	0.470
Esophagus	Dmean (Gy)	19.45	10.79	18.24	13.65	15.77	32.93	18.47±7.74	15.5	12.51	40.5	20.08	17.54	33.75	23.31±11.19	0.400
	Dmax Gy	6.93	5.89	9.1	5.99	5.86	11.96	7.62±2.46	5.67	3.4	10.71	4.3	3.3	7.62	5.83±2.89	0.270
Spinal Cord	Dmax Gy	14.2	16.09	11.65	10.67	36.6	14.98	17.37±9.64	11.77	22.25	14.05	13.79	11.38	19.17	15.40±4.36	0.040

There was no statistically significant difference in CI values between SIRA and DIRA plans. The CI of the SIRA plan was 0.9681±0.01, while that of the DIRA plan was 0.9721±0.01 with a p-value of 0.505. However, the SIRA planning produced a better homogeneity for bilateral whole breasts/bilateral chest wall irradiation than the DIRA planning. SIRA planning showed superior homogeneity with HI values of 0.0999±0.01 compared to DIRA planning with HI values of 0.1640±0.12 (p=0.230). The V95% of SIRA and DIRA plans were not significantly different, with mean values of 96.81±0.6% and 97.21±1.34%, respectively. The volume receiving 107% of the prescribed dose was less in both groups.

## Discussion

SBBC is a rare condition. There are no standard guidelines for its treatment till now, and due to the increased need for breast-preserving treatment, bilateral irradiation is required. The enormous and complex target volume and the necessity to minimise the dose to the heart and lungs make synchronous bilateral breast/chest irradiation difficult. BBCs have a large C-shaped target volume that can vary substantially in shape and volume. Furthermore, the target is closer to the skin, and OARs with large volumes are irradiated.

The 3DCRT-based radiation therapy strategy for BBCs has significant shortcomings, including insufficient target coverage and inhomogeneous dose distributions. Yusoff and colleagues compared 3DCRT and IMRT treatment strategies for BBC patients. They found that whereas both treatment strategies provided equivalent PTV coverage, IMRT outperformed OAR dose distribution to the lungs and heart [11]. Modern radiation therapy advancements like IMRT, VMAT, and helical tomotherapy enable us to treat SBBC patients with improved target dose uniformity and OAR sparing. Several studies have discussed which radiation technique is the best for SBBC irradiation according to the dosimetric characteristics [12].

In the current investigation, Arc treatment was employed. According to the QUANTEC study, the incidence of radiation pneumonitis is less than 20% if V20Gy<30% and V30Gy< 15% with conventional fractionation [12]. In this study, both the right and left lungs achieved the dose constraints, with the mean dose received by the right lung in SIRA and DIRA plans being 11.22±2.19 Gy and 12.04±2.96 Gy, respectively. The mean dose received by the left lung is 11.24±1.96 Gy and 12.12±2.06 Gy, respectively. The V5Gy, V10Gy and Dmean of the heart and its chambers and LAD doses were also compared between the two plans. The mean LAD dose of SIRA was higher than that of DIRA planning.

In an earlier study on SBBC, Banaei et al., who compared the mono isocentric (MIT) and dual isocentric techniques (DIT) in chest wall radiation therapy of mastectomy patients, showed that there was no significant difference between the two radiotherapy planning techniques regarding dose distribution in the OARs and the 95% of the prescribed dose coverage of the target tissue. However, the maximum dose delivered by 107% of the prescribed dose coverage was higher in DIT. Therefore, they recommended MIT instead of DIT for better conformal radiotherapy [12].

The number of arcs in RA planning is acknowledged to influence the results of treatment plans significantly. According to a 2009 study by Nicolini et al. [13], two half-arc RA plans provide better dose distribution than one or two full-arc therapy plans.

Bilateral lungs are invariably exposed to more radiation in arc technique when both breasts are treated simultaneously. Subramanian et al. suggested the hybrid VMAT technique to address this issue. However, this approach is only used for SBBC and does not include regional lymph node irradiation [14].

Motion control and breath control were not used in this investigation. Breath control for 3DCRT is now available in our institute, but not for RA. Previous studies have established the mid-ventilation phase as a suitable substitute for breath control because it is the phase during which targets can be "seen" by static beams for the most extended duration when proper margins are defined [15].

When planning RT for SBBC, Kim et al. noted that it was challenging to determine the precise dose from two different isocentres in some treatment planning systems [5]. Apart from the dosimetric aspects, the SIRA technique requires less setup time as it involves setting up the patient at the isocentre, setup verification using imaging, implementing setup correction (if any) and treatment. For the DIRA plans, these steps must be performed sequentially for each isocentre. Hence, the overall patient-in to patient-out time will be more in the DIRA approach.

The table below summarises studies on SBBC with regard to lung and heart doses (Table 3).

**Table 3:** List of previous studies on synchronous bilateral breast cancer demonstrating lung and heart doses DIRA: dual Isocentric RapidArc; SIRA: single isocentric RapidArc

Title	Author	Heart Dmean(Gy)	Total lung Dmean (Gy)	Reference
Radiation therapy of synchronous bilateral breast carcinoma (SBBC) using multiple techniques	Kim et al., 2018	14.47	15.84 (left), 18.32(right)	[5]
First clinical report of helical tomotherapy with simultaneous integrated boost for synchronous bilateral breast cancer	Wadasadawala et al., 2015	4.68	5.99	[16]
Bilateral breast irradiation using hybrid volumetric modulated arc therapy (h-VMAT) technique: a planning case report	Subramanian et al., 2016	10.5,16.2	12.1,13.8	[14]
Synchronous bilateral breast cancer irradiation: clinical and dosimetrical issues using volumetric modulated arc therapy and simultaneous integrated boost	Fiorentino et al., 2017	5	9.3	[17]
Radiation therapy of synchronous bilateral breast carcinoma (SBBC) using volumetric modulated arc therapy (VMAT)	Wang and Park, 2015	10.3	10.3	[18]
Dosimetric comparison of coplanar versus non-coplanar volumetric modulated arc therapy for treatment of bilateral breast cancers	Bharati et al. 2023	13.9	7.28	[19]
Cardiac dose in the treatment of synchronous bilateral breast cancer patients between three different radiotherapy techniques (VMAT, IMRT, and 3D CRT)	Salim et al. 2023	3.99	7.36,8.09	[20]
Present study		6.03±0.52 (SIRA) 7.28±1.38 (DIRA)	SIRA= Left (11.24±1.96); Right (11.22±2.19) DIRA= Left 12.12±2.06); Right (12.04±2.96)	

The study's limitations include the small number of patients, which is due to the rarity of SBBC. The clinical follow-up of the cases, as well as the daily setup variations, have not been documented. 

## Conclusions

This study reports the cardiac dose sparing (heart and its chambers and left anterior descending artery) and compares the SIRA technique with the DIRA technique. The SIRA planning produced a better homogeneity for bilateral whole breast/bilateral chest wall irradiation than the DIRA planning. In SIRA planning, the setup time and the overall machine time are less. Advanced long-term follow-up studies with a large number of patients are required to determine the clinical efficacy of the RA programmes in terms of oncologic outcomes and treatment toxicities.
